# Transplantation

**Published:** 1877-05

**Authors:** 


### TRANSPLANTATION.

I report to the profession a case of transplantation, which after the space of seven weeks gives every evidence of success. The tooth is firm, and the jaws surrounding perfectly healthy. By the closest examination the transplanted tooth cannot be detected.

The patient is a young man twenty-one years of age, with an excellent constitution. The tooth is an upper left lateral, and was taken from the mouth of a lady sixty years of age. With the dental engine, I drilled the pulp cavity, and filled it with gold. In doing this, almost a half hour passed before the tooth was inserted. Sufficient time was this, for the tooth to become cold.

I think the result of the case depended much upon the plan I adopted to hold the tooth in place, while union was taking place. In connection with a silk thread, I used a strip of rubber dam. This strip was about one-fourth of an inch long, and one-eighth of an inch wide, with two holes in each end. The center of the strip was placed over the cutting surface of the tooth the two ends looking upward. The thread was wound around the adjoining teeth near the gums, passing through the holes in the rubber, and the thread tied so as to make a gentle and steady pressure on the tooth. This was left one week without pain, and but little inconvenience. I used no treatment except the application of Tincture of Arnica. The tooth, for some time, was kept entirely free from use.

I do not regard the transplanting a tooth any more remarkable than replanting. It may require a longer time for the former to become firm, as new bone must be deposited to fill up any irregularities, or rather to make a new socket for the new tooth.

I was especially thanked by the father for refusing to tell the son where the tooth came from, as it would have been a source of annoyance, and he had no business knowing.

